# Dual-Triggered Release Mechanisms in Calcium Alginate/Fe_3_O_4_ Capsules for Asphalt Self-Healing: Cyclic Load-Induced Sustained Release and Microwave-Activated On-Demand Delivery

**DOI:** 10.3390/polym17233187

**Published:** 2025-11-29

**Authors:** Pei Wan, Jiazhu Wang, Zirong Ma, Zhiming Lin, Peixin Zhong, Xiaobin Zou, Yilun Shen, Niecheng Lin, Hang Chen, Shaopeng Wu, Quantao Liu, Jianlin Feng, Lei Zhang, Xing Gong

**Affiliations:** 1 Fujian Provincial Transportation Research Institute Co., Ltd., Fuzhou 350004, China; wanpei@fjits.net (P.W.); wangjiazhu@fjits.net (J.W.); mazirong@fjits.net (Z.M.); linzhiming@fjits.net (Z.L.); zhongpeixin@fjits.net (P.Z.); zouxiaobin@fjits.net (X.Z.); shenyilun@fjits.net (Y.S.); linniecheng@fjits.net (N.L.); 2 Superior College for Engineers, Chang’an University, Xi’an 710064, China; 3 State Key Laboratory of Silicate Materials for Architectures, Wuhan University of Technology, Wuhan 430070, China; liuqt@whut.edu.cn (Q.L.); fengjianlin@whut.edu.cn (J.F.); 4 Wuhan University of Technology Advanced Engineering Technology Research Institute of Zhongshan City, Zhongshan 528400, China; 5 School of Materials Science and Engineering, Chang’an University, Xi’an 710064, China; lei.zhang@chd.edu.cn; 6 Research Institute of Highway Ministry of Transport, Beijing 100088, China; x.gong@rioh.cn

**Keywords:** calcium alginate/Fe_3_O_4_ capsules, cyclic load, microwave irradiation, rejuvenator release

## Abstract

The calcium alginate/Fe_3_O_4_ capsules with multi-chamber structure can release interior rejuvenator under cyclic load and microwave irradiation; however, the rejuvenator release mechanism of capsules under two types of external activation is still unknown. Hence, this paper investigates the rejuvenator release mechanism of capsules in asphalt concrete under cyclic load and microwave irradiation. This research covers the synthesis of calcium alginate/Fe_3_O_4_ capsules and the evaluation of fundamental characteristics. The asphalt concrete containing capsules are subjected to cyclic load and microwave irradiation, respectively. The rejuvenator discharge ratio of capsules after external activation is determined using FTIR spectrum analysis. Furthermore, the structure characteristics of the extracted capsules are monitored after cyclic load and microwave irradiation. The findings indicate that the capsules present a sustained release feature under cyclic load. The outer capsule surfaces forms microcracks (diffusion channel) and inner chamber walls generate micropores (release channel) under cyclic load pressure. The capsules release inner rejuvenator rapidly under the microwave irradiation. The nano-Fe_3_O_4_ particles generate irregular movement and form microwave action spots under the action of microwave irradiation, and the micropores (release and diffusion channel) occur on the outer surface of capsules and inner chamber wall. This paper reveals the mechanism of long-lasting slow release under cyclic load and active release under microwave irradiation of dual-responsive capsules, which may provide a theoretical basis for the all-season service of the capsule and the long-term intelligent maintenance of asphalt pavement.

## 1. Introduction

Bitumen concrete is widely selected as a paving material in high-grade and urban pavement due to its exceptional performance and ease of maintenance [1,2]. However, as service time rises, microcracks and aging are unavoidable in asphalt concrete due to prolonged exposure in natural surroundings and constant vehicle load [3,4,5,6]. If timely pavement repair is not applied, microcracks will progress into macrocracks and the degree of aging will worsen. This will have a negative impact on traffic safety and result in a reduced service life of the pavement. The existing crack treatment techniques, including slurry seal [7], fog seal [8], chip seal [9], crack sealing [10], micro-surfacing [11] and ultra-thin overlay [12], are remedial treatment methods that are carried out after extensive damage has already occurred. These methods fail to effectively tackle the matter of micro-damage in the pavement during the early service period [5,13]. In addition, these maintenance activities are marked by substantial expenses and considerable ecological damage, which goes against the road’s dedication to environmentally conscious maintenance techniques [14,15,16]. Therefore, it is necessary to implement an efficient maintenance treatment that focuses on repairing microcracks in the early stages and renewing aging asphalt during the service period in a low-carbon maintenance background.

Asphalt typically exhibits viscoelastic behavior in its natural environment and hence possesses inherent self-healing capabilities [17,18,19]. However, the speed at which the asphalt binder undergoes self-repair throughout its service lifespan is significantly slower than the speed at which cracks develop in asphalt pavement [20,21]. Hence, it is imperative to devise a maintenance strategy that efficiently enhances the self-repairing capacity of asphalt.

Recently, self-healing capsules containing a healing agent that follows the notion of composition balance have offered a new solution for the prompt mending of microcracks and the rejuvenation of aged asphalt [22,23,24]. The self-healing capsules consist of microcapsules (μm size, core–shell structure) [25,26,27,28] and capsules (mm size, multi-chamber structure) [29,30,31,32]. The microcapsules can rupture the outer shell and release their inner healing agent when subjected to stress at the tip of a microcrack, which accelerates the crack repair process. However, the mode in which microcapsules burst is mainly dependent on the level of compatibility between the resilience of the capsule and the tip stress applied to the crack. When the stress on the crack is lower than the strength of the capsules, the microcrack will avoid the microcapsules, preventing the release of the encapsulated rejuvenator. The evident disadvantage of unregulated rejuvenator discharge limits its use in asphalt concrete. The multi-chamber calcium alginate capsules can discharge their interior rejuvenator gradually via elastic contraction–expansion (similar to the contraction–recovery of sponge before and after stress) when subjected to external traffic load without causing noticeable harm to the capsule shell. The calcium alginate capsules have a sustained release feature and provide prolonged healing function for asphalt concrete [33,34].

The existing capsules’ rejuvenator release is a passive and unpredictable mode triggered by stress (crack tip stress or cyclic load stress). However, this stress-induced release strategy does not guarantee the prompt release of the healing agent and fails to give the necessary amount of healing agent when required. Controlled release of rejuvenator contained within responsive capsules in asphalt materials can be achieved by using external stimuli such as a magnetic field and microwave, considering the service conditions of the capsules [35]. The multi-responsive healing system, equipped with responsive capsules, may accomplish both gradual and rapid release characteristics in response to different environmental stimuli. In the previous research, the nano-Fe_3_O_4_ was successfully inserted in calcium alginate capsules [36], and the Ca-Alginate/Fe_3_O_4_ capsules can release inner rejuvenator under the action of cyclic load and microwave irradiation [37,38], and the asphalt concrete with dual-responsive capsules shows superior healing potential in different service environments [39]. However, the mechanism of rejuvenator release from dual-responsive self-healing capsules has not been clarified in detail, which has hindered the optimization of the material design of the capsules and the quantitative assessment of the healing capacity of asphalt concrete.

The internal chamber structure of the capsule determines the release pattern of the rejuvenator. In view of this, this paper explores the release mechanism of Ca-alginate/Fe_3_O_4_ capsules under the action of cyclic load and microwave irradiation. First, an ideal model of the capsule’s internal chambers (pomegranate-like structure) is established. Second, the release rate of the regenerative agent under different excitation conditions is quantitatively characterized. The morphological structure of the capsule after loading and microwave irradiation is systematically observed. Finally, the dual-release mechanism of the capsule is elaborated in detail.

## 2. Materials and Methods

### 2.1. Materials

Table 1 shows essential information about the materials utilized to produce the calcium alginate/Fe_3_O_4_ capsules. This research focuses on using sunflower oil as an asphalt rejuvenator. Table 2 provides an overview of the fundamental characteristics of sunflower oil. Sunflower oil has the ability to rejuvenate deteriorated bitumen [40,41] and exhibits a distinct absorption peak between 1740 and 1750 cm^−1^ in the FTIR spectrum, while the virgin asphalt shows no absorption peak [42,43]. Therefore, the amount of sunflower oil released from the capsules can be ascertained via FTIR spectrum. In this research, the base 60/80 bitumen is used to prepare asphalt mixtures. The bitumen’s density, penetration, softening point, and ductility are measured to be 1.037 g/cm^3^, 68.6 (0.1 mm), 48.5 °C, and 183 cm (15 °C, 5 cm/min), respectively.

### 2.2. Preparation of Calcium Alginate/Fe_3_O_4_ Capsules

**Figure 1:**
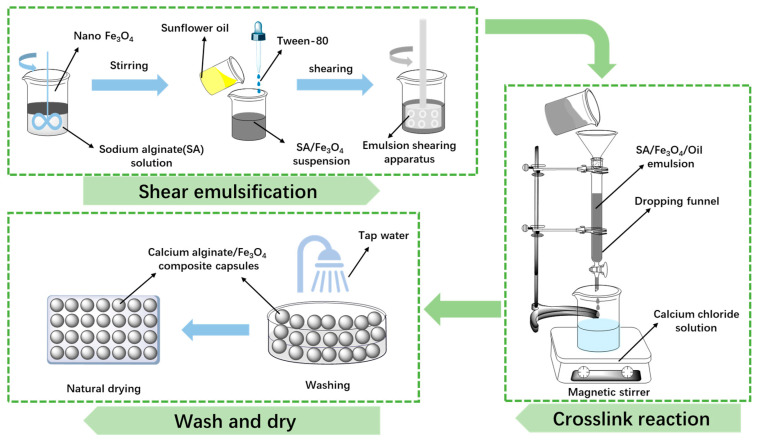
The production procedure of Ca-alginate/Fe_3_O_4_ capsules [37].

**Figure 2:**
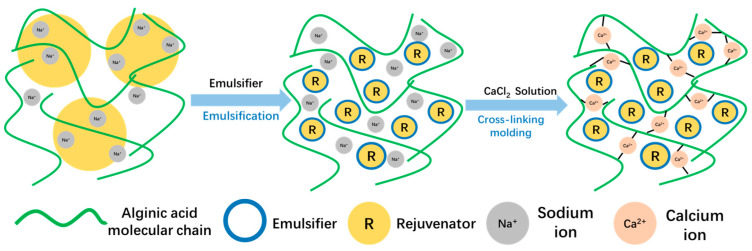
The formation mechanism of calcium alginate capsules.

The method for fabricating Ca-alginate/Fe_3_O_4_ capsules is presented in Figure 1. The process can be divided into three stages: (1) shear emulsification, (2) cross-linking reaction, and (3) wash and dry. The precise synthesis procedure is stated in prior studies [36,37,38,39]. The formation mechanism of calcium alginate capsules is shown in Figure 2.

### 2.3. Characterization of Prepared Capsules

Multiple investigations are carried out on the fabricated capsules to ascertain their essential functionality. The morphological structure of capsules is examined using scanning electron microscopy (SEM) (Zeiss, Oberkochen, Germany). In addition, the mechanical resistance of capsules is assessed using the uniaxial compression test (UCT). The thermal sensitivity characteristic of capsules is explored via a concurrent thermal analyzer. Table 3 presents the essential specifications of the testing equipment.

### 2.4. Production of Bitumen Concrete with Capsules

The work employs a dense bitumen mixture, and the gradation (AC-13) is displayed in Table 4. During the last step of the mixing process, the capsules are mixed into the bitumen concrete. The mass of added capsules is 0.5% of the whole quantity of the bitumen mixtures [44,45,46]. After mixing, the rutting plate molding equipment is utilized to produce bitumen mix plates with capsules. The beams with dimensions of 98 mm × 45 mm × 50 mm are extracted from the bituminous concrete plates. A notch measuring 5 mm × 4 mm is created in the middle.

### 2.5. External Activation for Bitumen Concrete with Capsules

**Figure 3:**
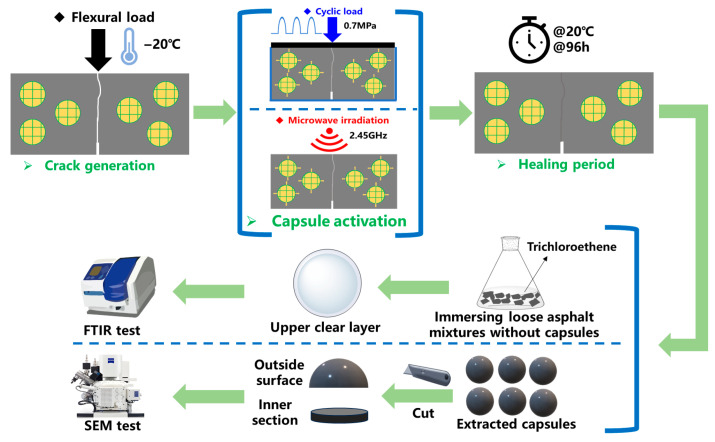
The activation procedure of bitumen concrete containing capsules.

**Figure 4:**
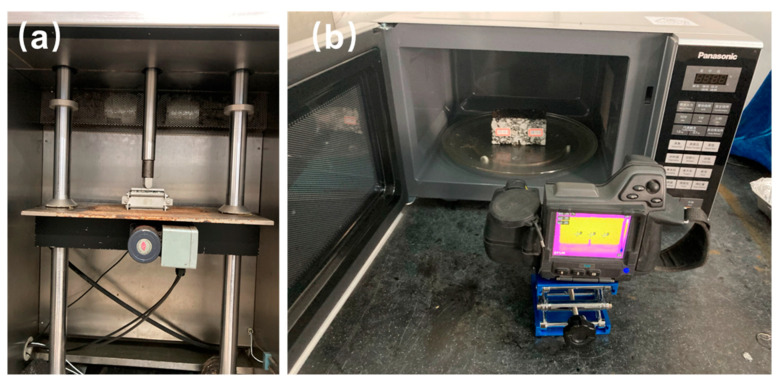
Two types of external activation for asphalt concrete with capsules: (**a**) cyclic load and (**b**) microwave irradiation.

The multi-chamber capsules need to be activated to release the inner rejuvenator via external medium. In this work, two types of external activation (cyclic load or microwave irradiation) are conducted on the beams with capsules through a universal testing machine and microwave oven, respectively. The specific test procedure is presented below:

**(1) Cyclic load for asphalt concrete with capsules**. As depicted in Figure 3, the concrete beams containing capsules are placed in a thermostat at −20 °C for 8 h. Subsequently, the 3PB tests are performed on the concrete beams to produce observable cracks. The initial speed of the load is set to 0.5 mm/min. The fractured beams containing capsules are placed within the steel mold and subsequently exposed to repeated load (as presented in Figure 4a). The steel plate is placed on the beams to distribute the load pressure evenly. The test beams undergo compression loads (0.7 MPa) of 16,000, 32,000, 48,000, 64000, 96,000, and 128,000 cycles, respectively, at 20 °C. The load frequency is set as 1 Hz. Following the application of cyclic load, the test beams are then placed in a thermotank set at 20 °C for a period of 48 h to restore their structural integrity.

(2) **Microwave irradiation for asphalt concrete with capsules**. The crack generation procedure in this section is the same as the procedure in the above section (1). After the occurrence of cracks, the fractured beams are put in a microwave oven and subjected to microwave irradiation (as presented in Figure 4b). The irradiation time is 30 s, 60 s, and 90 s, and the work frequency of the microwave oven is 2.45 GHz. After irradiation, the healing condition is the same as the procedure in the cyclic load section.

### 2.6. Characterization of the Internal Structure and Rejuvenator Discharge Degree of Capsules After Cyclic Load and Microwave Irradiation

**Figure 5:**
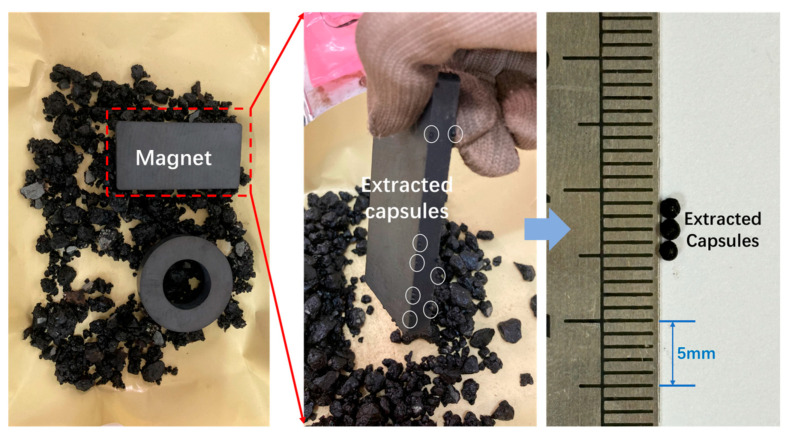
The magnetic separation of capsules in loose asphalt mixtures.

The healed asphalt concrete beams following cyclic load and microwave irradiation are put in the oven at 80 °C for 30 min to obtain loose asphalt mixtures. The capsules in the asphalt mixtures are later removed via the magnets and manual extraction, as shown in Figure 5. The extracted capsules are cleaned to remove the asphalt. Then the capsules are cut into hemispherical and interfacial samples, and then the SEM test is performed on the prepared samples to observe the outer and inner structure.

The remaining bitumen mixtures are dissolved in a C_2_HCl_3_ solution for a duration of 48 h. The supernatant is subsequently transferred to a fuming drawer and left undisturbed for a duration of 24 h to promote the evaporation of solvent. A centrifuge tube is filled with 0.1 g of bitumen, followed by the addition of 2 mL of CS_2_ to disperse the bitumen. Bitumen, along with released oil, is applied onto KBr wafers and subsequently dried to form a bitumen film. The FTIR tests are performed in the mid-infrared spectral range, specifically covering wavenumbers ranging from 400 to 4000 cm^−1^. The field parameter is set to a width of 4 cm^−1^, and the total test time is defined as 64.

**Figure 6:**
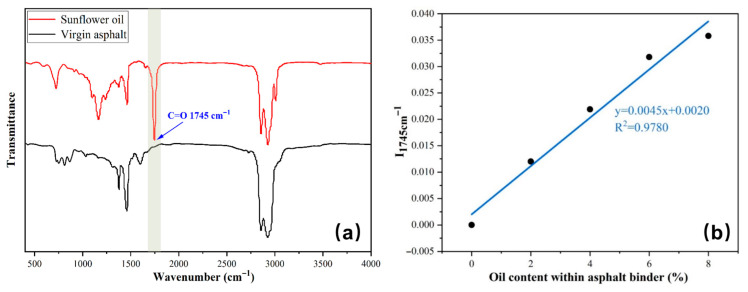
(**a**) The infrared spectrum of virgin asphalt and rejuvenator, and (**b**) the relationship between I_1745 cm_^−1^ and the rejuvenator proportion within bitumen [37,38,47].

Figure 6a illustrates that the infrared spectra of sunflower oil exhibit a prominent peak at a wavenumber of 1745 cm^−1^. On the contrary, virgin bitumen does not exhibit any absorption value within this range.

Figure 6b depicts the relationship between the index I_1745 cm_^−1^ and the rejuvenator concentration in the bitumen binder. The rise in the index I_1745 cm_^−1^ is directly proportional to the amount of oil in the bitumen binder (y = 0.0045x + 0.0020, R^2^ = 0.9780). Hence, the percentage of oil present in the bitumen binder obtained from mixed beams can be ascertained by examining the index I_1745 cm_^−1^ of different asphalt binders.

$$I_{{1745cm}^{-1}}=\frac{The\;peak\;area\;of\;1745\;{cm}^{-1}}{\sum Area\;of\;spectral\;bands\;between\;2000\;and\;600\;{cm}^{-1}}$$

After subjecting the beams to loading or microwave treatment, we processed them to remove capsules and subsequently extracted the asphalt from the beams. Subsequently, the extracted asphalt undergoes infrared spectroscopy testing to calculate the characteristic peak index, which is then used to determine the rejuvenator content within the beam asphalt. Finally, the rejuvenator release rate of the capsules is defined by calculating the ratio of the rejuvenator content in the extracted asphalt after cyclic load or microwave irradiation to the total amount of rejuvenator encapsulated within all capsules in the beam.

## 3. Results and Discussion

### 3.1. Fundamental Properties of Prepared Ca-Alginate/Fe_3_O_4_ Capsules

**Figure 7:**
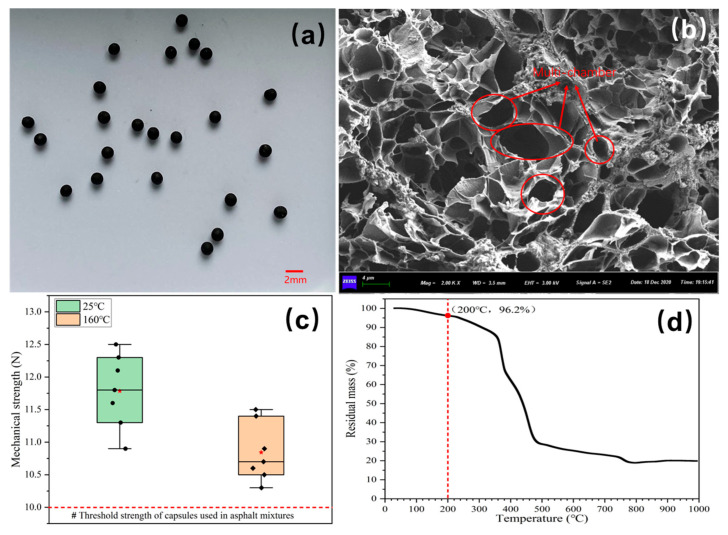
The basic information about the prepared capsules [39]: (**a**) morphologic appearance, (**b**) interior structure, (**c**) mechanical strength (Black squares: measured values, red asterisks: average values), and (**d**) thermal resistance [39].

Figure 7a and Figure 7b show the physical features and inner structure of the prepared capsules, respectively. The capsules have a roughly spherical shape, and the inner structure presents complicated multi-chamber organization. The rejuvenator, sunflower oil, is stored in distinct chambers of varying sizes and architectures. The capsule’s unique storage pattern enables the gradual release of its healing components upon exposure to external pressure.

Figure 7c displays the mechanical strength of the prepared capsules (Black squares represent measured values, while red asterisks denote average values.). The mm sized capsules are designed for use in asphalt mixtures as fine aggregate elements. The capsule must have sufficient mechanical strength to resist the effects of stress during asphalt concrete preparation. An experimental study demonstrates that the capsules used in bitumen mixtures need a mechanical strength of more than 10 N [48]. The Ca-alginate/Fe_3_O_4_ capsules exhibit a yield strength of 11.8 N, which demonstrates that their mechanical ability meets the preparation requirements of asphalt concrete.

The capsules are added to bitumen mixtures in the last mixing procedure and are then subjected to compression molding. The capsules must survive the high temperatures generated during the production process. Hence, the temperature at which calcium alginate capsules disintegrate thermodynamically should be greater than the production temperature of bitumen mixtures. Figure 7d presents the mass loss curve of capsules. The capsules have a mass loss of 3.8% at a temperature of 200 °C. Therefore, the capsules demonstrate exceptional temperature resistance throughout the asphalt concrete manufacturing process.

### 3.2. Rejuvenator Discharge Ratios of Capsules After Two Forms of Activation

The healing agent discharged from the capsules is critical for the healing of cracks in asphalt concrete. Therefore, this section quantitatively analyzes the discharge rate of the healing agent in the capsule under two types of external activation and explores the healing agent release patterns of the capsule under external stimulation.

#### 3.2.1. Rejuvenator Discharge Ratios of Capsules After Cyclic Load

**Figure 8:**
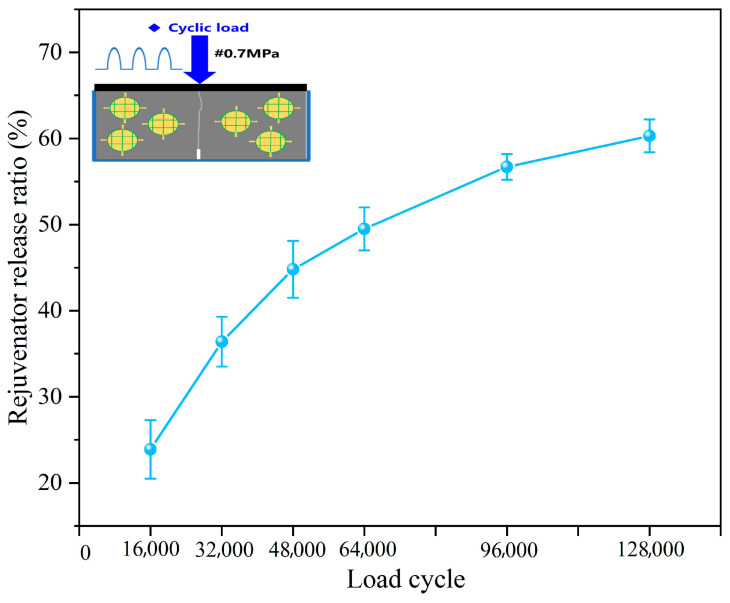
The rejuvenator discharge ratios of capsules in concrete beams after cyclic load [37].

Figure 8 presents the rejuvenator discharge degree of capsules in concrete beams with capsules after different cycles of load. After 16,000, 32,000, 48,000, 64,000, 96,000, and 128,000 cycles of load, the rejuvenator discharge ratios of capsules are 23.9% (±3.4%), 36.4% (±2.9%), 44.8% (±3.3%), 49.5% (±2.5%), 56.7% (±1.5%), and 60.3% (±1.9%), respectively. As the number of cyclic loads increases, the force response time of the capsule grows and the extrusion time of the internal chamber prolongs; hence, the release time of the rejuvenator increases, leading to a consequent increase in the rejuvenator release rate.

The results of the load-responsive release characteristics of the capsules show that the multi-chamber calcium alginate/nano-Fe_3_O_4_ capsules have the ability of long-lasting release. During cyclic load, the capsule does not release all the asphalt restorative at once but gradually releases the restorative encapsulated inside. Therefore, the capsule made in this paper is expected to realize the gradual release of the repair agent during the service period of the pavement through the cumulative effect of the pressure of the external driving vehicles and realize the long-lasting repair of asphalt concrete.

#### 3.2.2. Rejuvenator Discharge Ratios of Capsules After Microwave Irradiation

**Figure 9:**
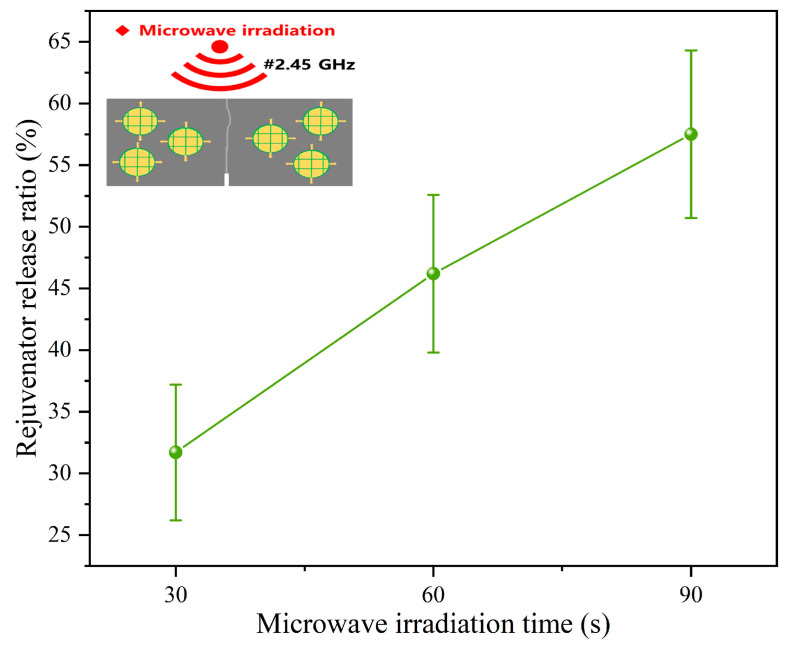
The rejuvenator discharge ratios of capsules in a concrete beam after microwave irradiation [37].

Figure 9 presents the rejuvenator discharge ratios of capsules within asphalt concrete following microwave exposure. After 30 s, 60 s, and 90 s of irradiation, the rejuvenator discharge ratios of capsules are 31.7%, 46.2%, and 57.5%, respectively. It indicates that with the extension of microwave exposure time, the amount of discharged rejuvenator increases. Contrasting with the gradual release characteristic of capsules under cyclic stress, the capsules can quickly release inner rejuvenator when subjected to short durations of microwave motion, which indicates that the capsule may provide a necessary amount of asphalt rejuvenator on demand.

### 3.3. The Structure Characteristics of Capsules Extracted from Bitumen Beams After Two Types of External Activation

#### 3.3.1. The Structure Feature of Capsules Extracted from Bitumen Beams After Cyclic Load

##### The Structure Feature of Outer Wall of Capsules Extracted from Bitumen Beams After Cyclic Load

**Figure 10:**
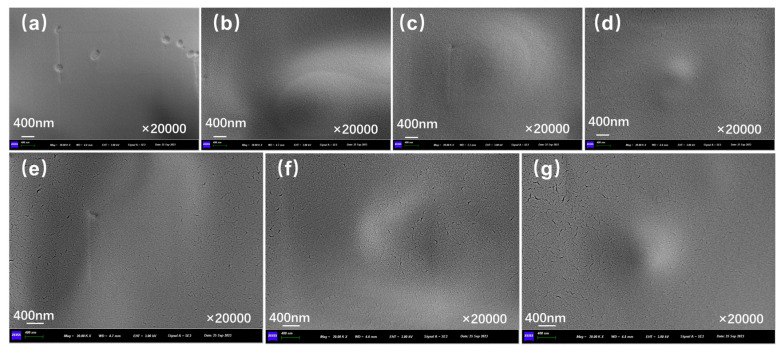
Outer surface morphology of the capsule after 0 (**a**),16,000 (**b**), 32,000 (**c**), 48,000 (**d**), 64,000 (**e**), 96,000 (**f**), and 128,000 (**g**) cycles of load.

Figure 10a presents the outer surface structure of capsules in asphalt concrete before and after cyclic load. The outer surface of the capsule remains intact overall, with no visible cracks present. The fine indentations on the capsule’s surface may have formed during the process of blending and compacting the asphalt mixture because the aggregates are pressed against each other. Figure 10b, Figure 10c, Figure 10d, Figure 10e, Figure 10f, and Figure 10g show the outer surface structure of capsules in asphalt concrete after 16,000, 32,000, 48,000, 64,000, 96,000, and 128,000 cycles of load, respectively. When the external cyclic load is applied to the concrete beams containing capsules, microcracks begin to appear on the outer surface of the capsule and the number of microcracks on the surface of the capsule increases as the extension of the load time, which indicates that the capsule will form nanoscale cracks on the outer surface under the extrusion of cyclic load and can form a diffusion channel for the repair agent.

##### The Structure Feature of the Internal Chamber of Capsules Extracted from Bitumen Beams After Cyclic Load

**Figure 11:**
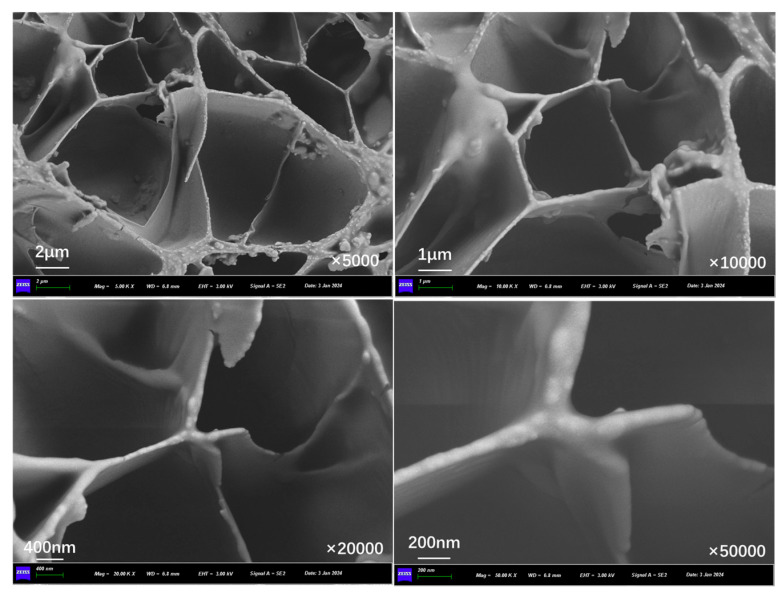
Morphological structure of the internal cross-section of the capsule without cyclic load.

Figure 11 presents the inner section structure of capsules in asphalt concrete without cyclic load. Without the effect of cyclic load, the interior of the capsule presents an obvious multi-chamber layout, the chambers are in a non-uniform distribution state, and there are no pores and cracks present on the chamber walls.

**Figure 12:**
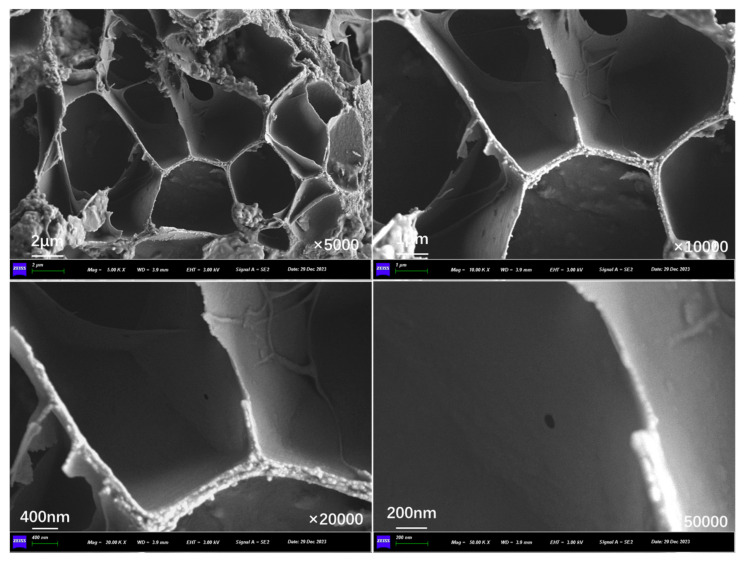
Morphological structure of the internal cross-section of the capsule after 16,000 cycles of load.

**Figure 13:**
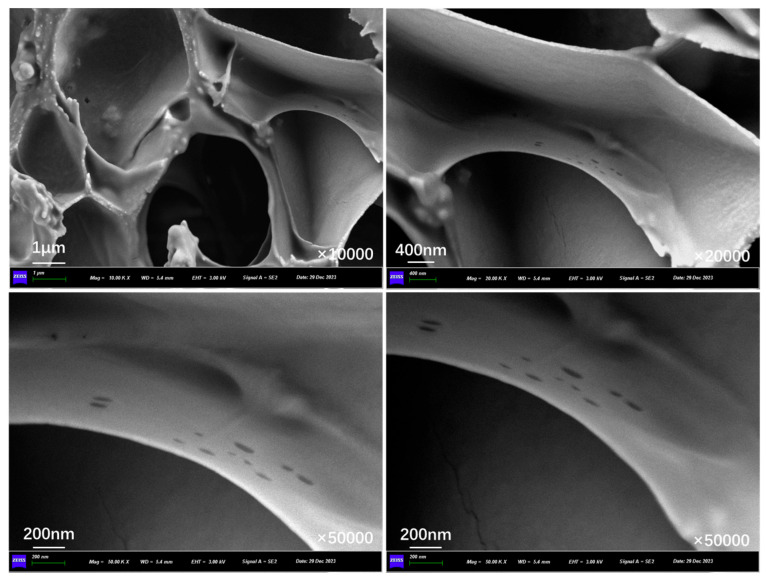
Morphological structure of the internal cross-section of the capsule after 32,000 cycles of load.

**Figure 14:**
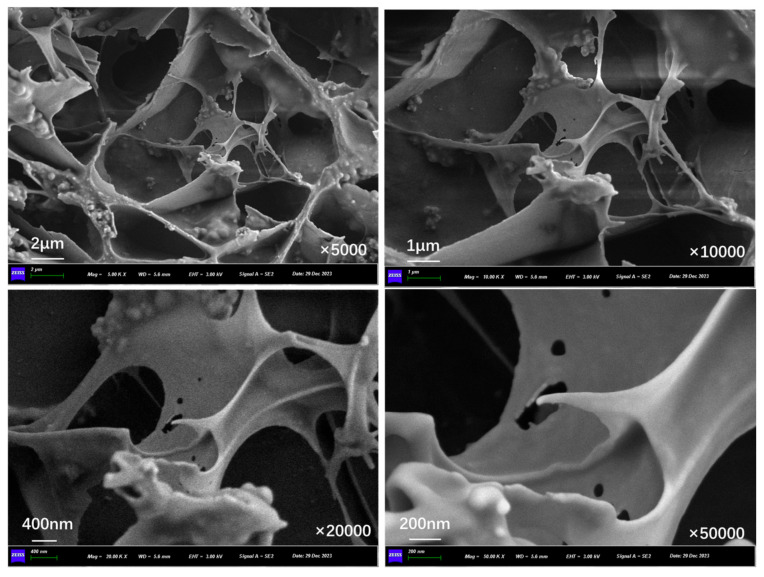
Morphological structure of the internal cross-section of the capsule after 48,000 cycles of load.

**Figure 15:**
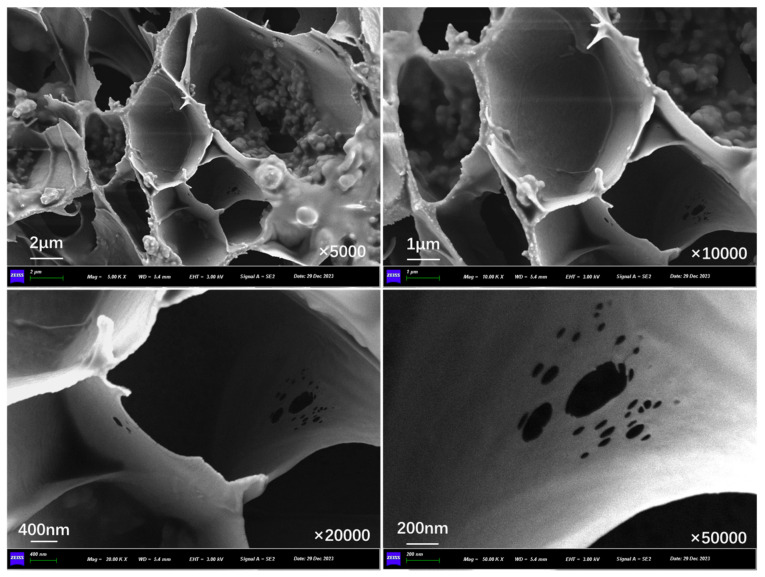
Morphological structure of the internal cross-section of the capsule after 64,000 cycles of load.

**Figure 16:**
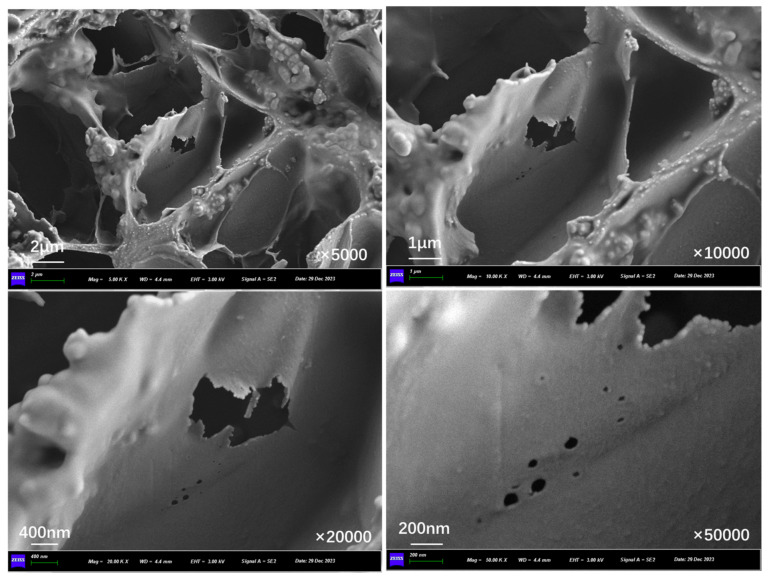
Morphological structure of the internal cross-section of the capsule after 96,000 cycles of load.

**Figure 17:**
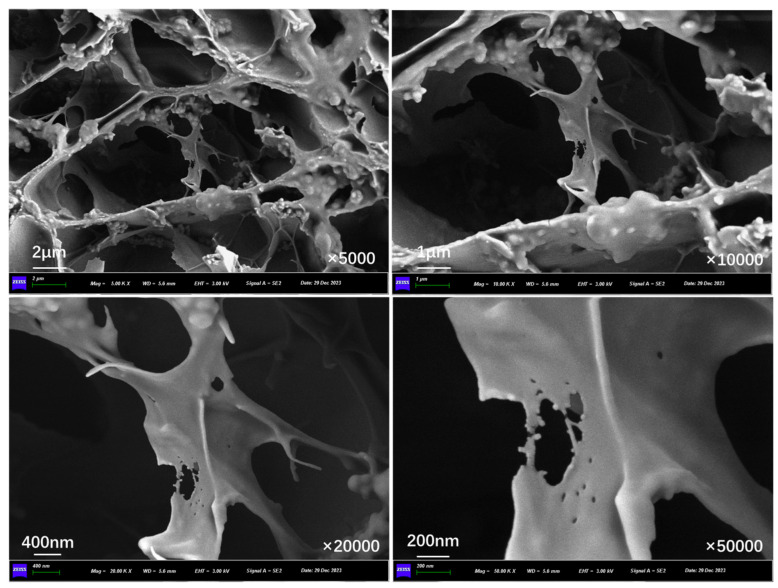
Morphological structure of the internal cross-section of the capsule after 128,000 cycles of load.

Figure 12, Figure 13, Figure 14, Figure 15, Figure 16, and Figure 17 show the inner section structure of capsules in asphalt concrete after 16,000, 32,000, 48,000, 64,000, 96,000, and 128,000 cycles of load, respectively. When the cyclic load is applied on the beam with capsules, obvious micropores appear on the internal chamber walls of the capsules, and the number of micropores on the walls of the chambers increases with the rise in load cycles, and the size of the pores becomes larger. The appearance of pores on the chamber wall is due to the continuous action of external load, where the chambers are squeezed against each other, and the deformation at the stress concentration is increased, leading to the generation of pores and micropores appearing on the chamber wall, which provide a channel for the release of the restorative agent.

#### 3.3.2. The Structure Features of Capsules Extracted from Bitumen Beams After Microwave Irradiation

##### The Structure Features of Outer Wall of Capsules Extracted from Concrete Beams Following Microwave Exposure

**Figure 18:**
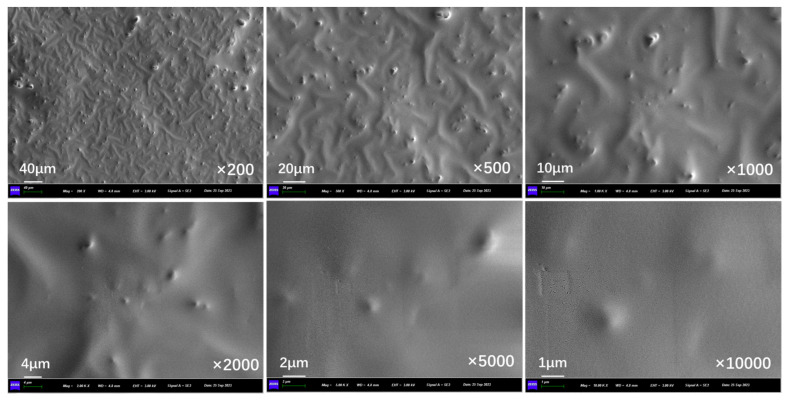
Outer surface morphology of capsules in asphalt concrete without microwave irradiation.

Figure 18 presents the outer surface structure of capsules in asphalt concrete without microwave irradiation. The outer surface of the capsule is slightly rough, the whole is in a closed state, and no pores exist on the surface.

**Figure 19:**
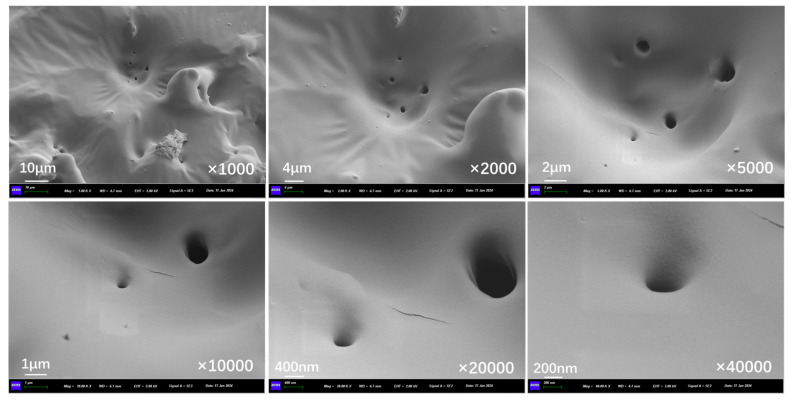
Outer surface morphology of capsules in asphalt concrete after third cycle of 10 s microwave irradiation.

**Figure 20:**
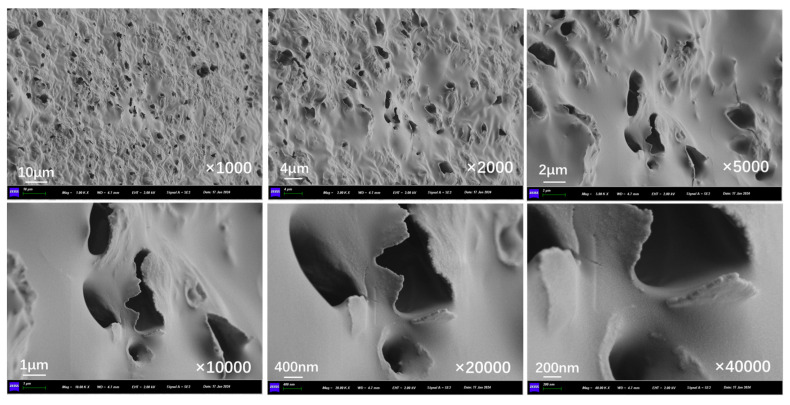
Outer surface morphology of capsules in asphalt concrete after third cycle of 20 s microwave irradiation.

**Figure 21:**
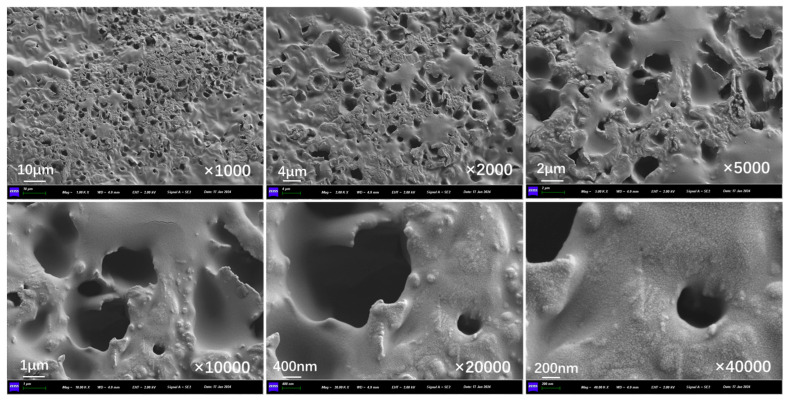
Outer surface morphology of capsules in asphalt concrete after third cycle of 30 s microwave irradiation.

Figure 19, Figure 20, and Figure 21 show the outer surface structure of capsules in concrete beams after 30 s, 60 s, and 90 s of microwave exposure, respectively. After microwave action, the outer surface of the capsules shows obvious pores. The calcium alginate capsule wall is mixed with nano-Fe_3_O_4_; when the microwave irradiation reaches the outer surface of the capsule, Fe_3_O_4_ nanoparticles in the alternating magnetic field appear in an irregular orientation of movement and will absorb the microwave heating to form a “hot spot”. Meanwhile, calcium alginate molecular chains will also be subjected to heat migration, so the capsule will form pores of different sizes on the outer surface of the capsule, providing a diffusion channel for the release of the restorative agent. In addition, it can be found that the number of pores on the outer surface of the capsule increases with the prolongation of the microwave action time because the longer the irradiation time, the more microwave “hot spots” are formed on the surface of the capsule, and the more restorative agent diffusion channels are formed.

##### The Structure Feature of Internal Chamber of Capsules Extracted from Concrete Beams After Microwave Exposure

**Figure 22:**
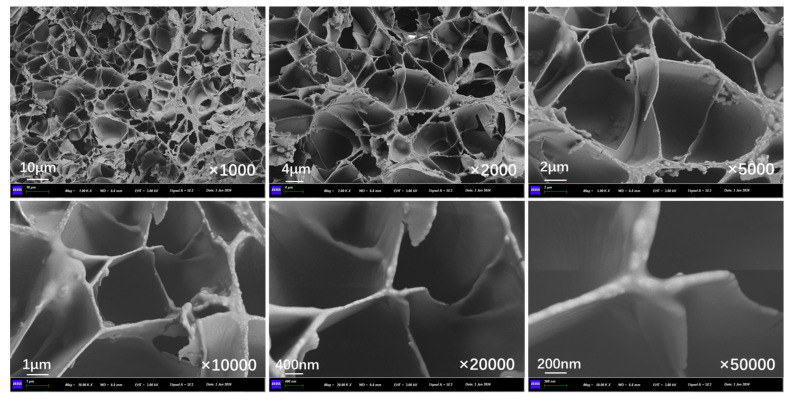
Internal cross-section morphological structure of capsules in asphalt concrete without microwave irradiation.

Figure 22 presents the inner section structure of capsules in concrete beams without microwave irradiation. In the absence of microwave irradiation, the interior of the capsules shows an obvious multi-chamber configuration, with the chambers in a non-uniform distribution, and no pores or cracks exist on the chamber walls.

**Figure 23:**
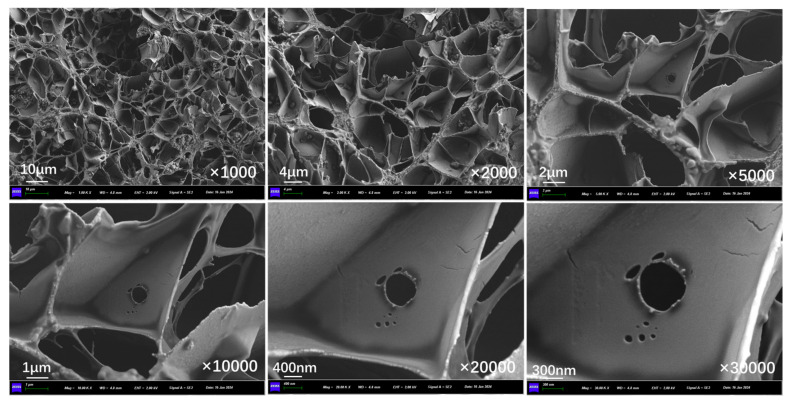
Internal cross-section morphological structure of capsules in asphalt concrete after third cycle of 10 s microwave irradiation.

**Figure 24:**
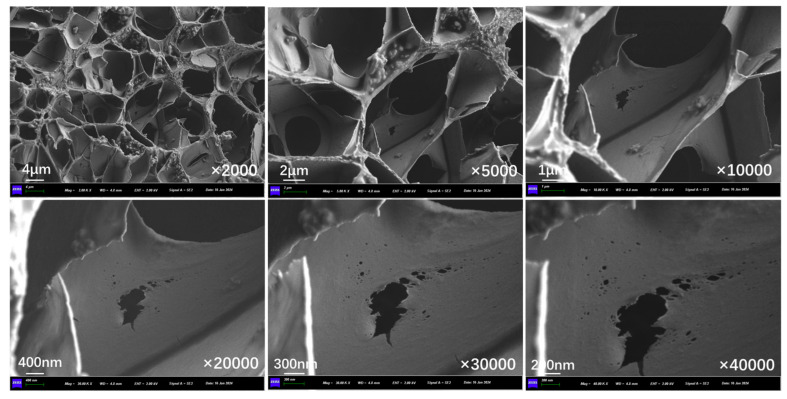
Internal cross-section morphological structure of capsules in asphalt concrete after third cycle of 20 s microwave irradiation.

**Figure 25:**
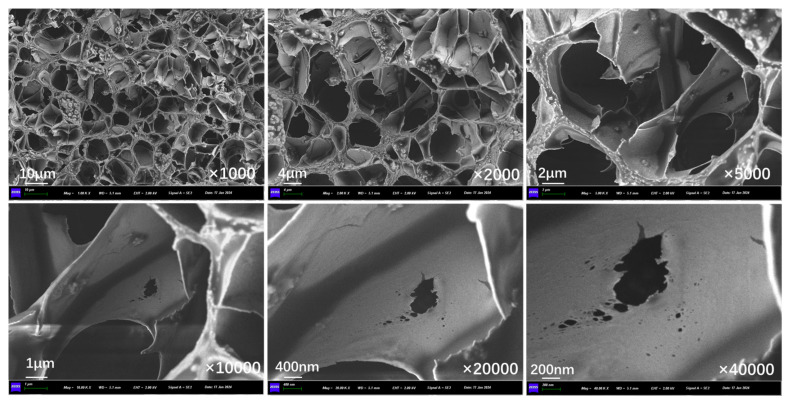
Internal cross-section morphological structure of capsules in asphalt concrete after third cycle of 30 s microwave irradiation.

Figure 23, Figure 24, and Figure 25 show the inner section structure of capsules in asphalt concrete after 30 s, 60 s, and 90 s of microwave exposure, respectively. When the capsule undergoes microwave irradiation, the chamber structure inside the capsule still exists, which indicates that the original special multi-chamber structure of the capsule will not disappear after microwave action, but pores of different sizes appear on the walls of the chambers. The microwave enters the capsule and is reflected between different chambers, and the nano-Fe_3_O_4_ particles in the chamber walls are subjected to an alternating magnetic field. The nano-Fe_3_O_4_ particles in the chamber wall undergo irregularly oriented motion in the alternating magnetic field and squeeze the chamber wall. At the same time, heat is generated, the chamber wall is heated to produce localized pores, and the restorative inside the chamber is released through the pores.

### 3.4. The Rejuvenator Discharge Mechanism of Capsules in Bitumen Concrete Under Two Types of External Activation

**Figure 26:**
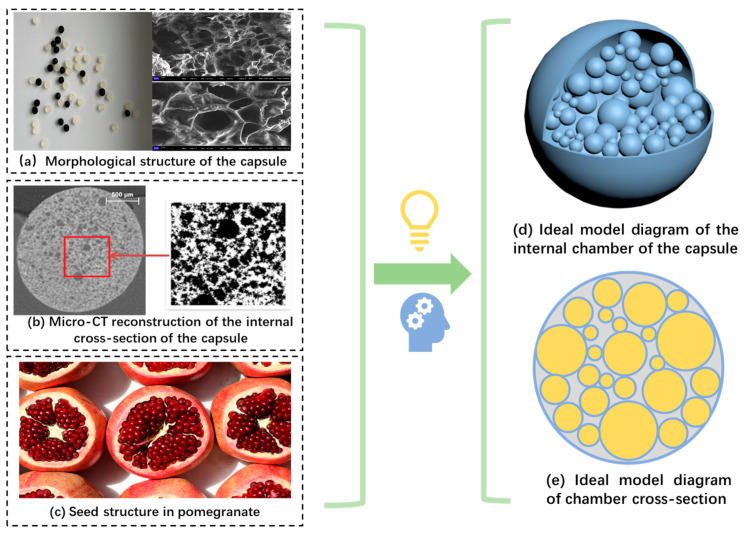
Model diagram of the internal chamber (**d**,**e**) of the capsule constructed from the morphology and internal structure (**a**) [38] (**b**) [49] of the capsule and pomegranate structure (**c**).

**Figure 27:**
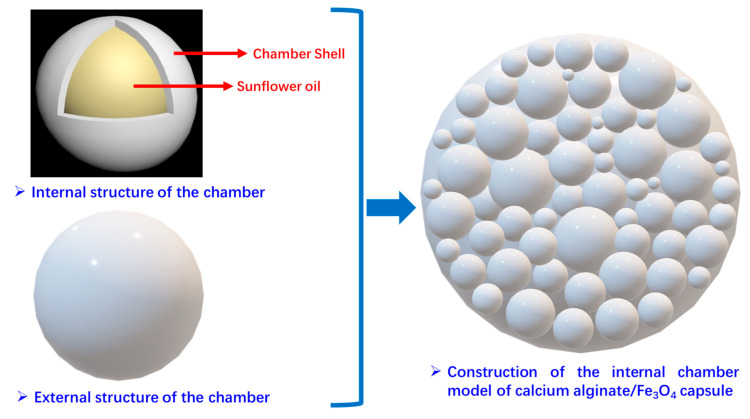
Construction of internal chambers in the capsule and chamber distribution model of the capsule.

As shown in Figure 26, from the macroscopic morphology and internal structure of the capsule and inspired by the spatial distribution of pomegranate seeds in the pomegranate, an ideal model of the internal chambers of the capsule is established accordingly in this paper. As presented in Figure 27 below, it is assumed that the chambers in the capsule are homogeneous spheres of different sizes, and many spherical chambers are randomly arranged in the capsule, and the chambers are adjacent to each other. The structure of individual chambers is characterized by a “core–shell structure”, in which sunflower oil is encapsulated in spherical chambers of different shapes, and the wall materials of the chambers are calcium alginate and nano-Fe_3_O_4_.

#### 3.4.1. The Rejuvenator Discharge Mechanism of Capsules in Asphalt Concrete Under Cyclic Load

**Figure 28:**
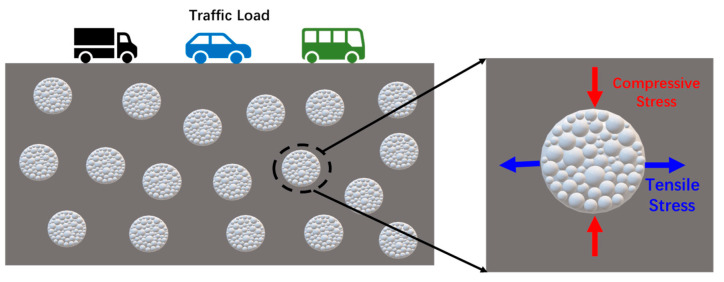
Force schematic of a single capsule in asphalt concrete as a traveling vehicle load passes over it.

**Figure 29:**
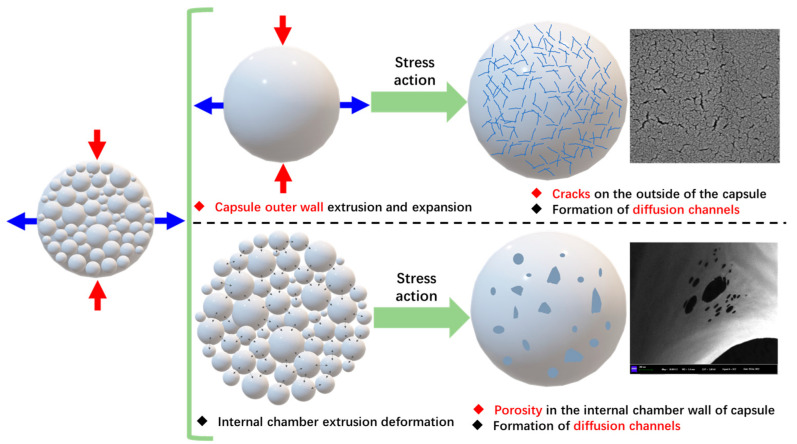
Schematic diagram of the load-induced release mechanism of capsules.

As shown in Figure 28, when the simulated load is applied to the asphalt concrete with capsules, the capsule is subjected to compressive and tensile stresses. The outer surface and inner chamber of capsules will generate structure variation under the action of stress. The load response release mechanism of the capsule is shown in Figure 29. The chambers inside the capsule are squeezed against each other, and the resulting stress concentration causes pores to appear in the chamber walls from which the repair agent is released. The outer wall of the capsule is subjected to the coupling of compressive and tensile stresses in the asphalt concrete, microcracks appear on the outer surface of the capsule, and the released restorative agent spreads out from the microcracks.

#### 3.4.2. The Rejuvenator Discharge Mechanism of Capsules in Asphalt Concrete Under Microwave Exposure

**Figure 30:**
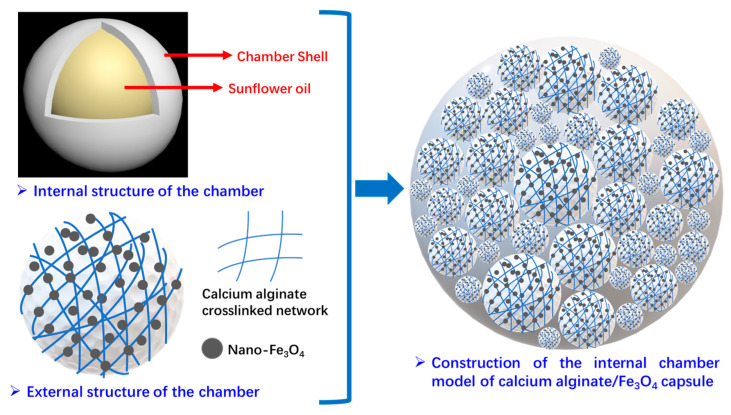
Construction of internal capsule chambers and distribution model of internal chambers of the capsule.

Based on the spherical chamber established in Section 3.4, the model of the internal chamber is refined in this section. As shown in Figure 30, it is assumed that the wall material of the chamber is a mesh structure composed of calcium alginate, and the nanoparticles of Fe_3_O_4_ are attached to the wall of the chamber to form a “target point” for microwave irradiation.

According to electromagnetic field theory, an electromagnetic wave is generated by the interaction of electric and magnetic fields, which propagates at the speed of light and perpendicular to each other’s direction of propagation. Therefore, during the propagation process of microwave, an alternating electromagnetic field is generated. When microwaves are applied to magnetic particles, a Lorentz force is generated on the particles, driving them into motion. In addition, the electromagnetic field may cause complex forms of motion such as rotation and vibration of the magnetic particles.

Hu et al. [50] embedded magnetic Fe_3_O_4_ in the double-layer capsule wall of PSS/PAH microcapsules and found that when the microcapsules were in a high-frequency alternating magnetic field, the pores with 50–100 nm appeared on the surface of the microcapsules, and the size of the pores increased with the extension of the excitation time of the magnetic field and eventually evolved into large cracks. The magnetic Fe_3_O_4_ particles in the microcapsules undergo exothermic phase transitions when subjected to the magnetic field, and the multilayer polyelectrolyte capsule wall is heated to produce pores. Long et al. [51] embedded the magnetic nano-Fe_3_O_4_ particles in the wall of the microcapsules, borrowed the principle of contraction and dilation of the heart, and applied an external magnetic field to the microcapsules. The nano-Fe_3_O_4_ particles in the capsule wall moved in the direction of the magnetic field strength to extrude the capsule wall, which led to a change in the permeability of the capsule wall, releasing the encapsulated core material. Upon removal of the magnetic field, the microcapsules reverted to their initial condition.

**Figure 31:**
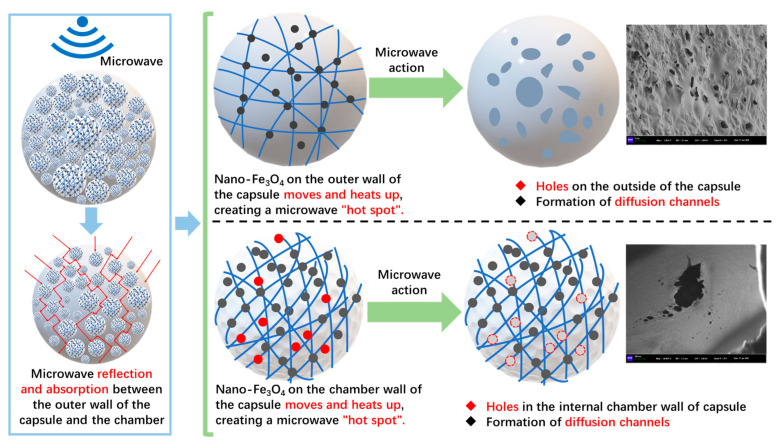
Schematic diagram of the microwave-induced release mechanism of capsules.

The microwave response release mechanism of the capsule is shown in Figure 31. When the microwave irradiation arrives at the capsule in the asphalt concrete, part of the microwave reflection occurs on the surface of the capsule and induces the magnetic nanoparticles of Fe_3_O_4_ on the wall of the capsule to rotate, vibrate, and other irregular movements. Meanwhile, the outer wall of the capsule generates microwave “hot spots”, leading to the emergence of micropores on the external surface of the capsule and the formation of the diffusion channel of the restorative agent. The microwave that enters the capsule reflects back and forth between the chambers, and the magnetic Fe_3_O_4_ nanoparticles undergo irregular orientation movement in the alternating magnetic field, squeezing the chamber wall and generating heat, which produces localized pores in the chamber wall, and the asphalt restorative inside the capsule chamber is released.

## 4. Conclusions

In this study, capsules are produced and their basic properties are assessed via a series of tests. The produced capsules are put into bitumen mixtures. The cyclic load and microwave irradiation are conducted on the bitumen concrete with capsules, respectively, to explore the rejuvenator release property. Furthermore, the structure variation characteristics of the extracted capsules are assessed following cyclic load and microwave exposure. Meanwhile, the rejuvenator release mechanism of capsules under two types of external activation is explained. The subsequent conclusions can be drawn:

1. The prepared Ca-alginate/Fe_3_O_4_ capsules possess multi-chamber organization. The capsules exhibit a mechanical strength of 11.8 N and experience a mass loss of 3.8% at 200 °C. These results satisfy the requirements of asphalt concrete manufacture.
2. The ideal chamber distribution in the capsule is established first. The chambers inside the capsule are uniformly shaped spheres of varying diameters, and several spherical chambers are haphazardly distributed within the capsule, with one chamber being close to another. The individual chambers exhibit a “core–shell structure”, where sunflower oil is enclosed inside spherical chambers of various forms. The chamber walls are composed of calcium alginate and nano-Fe_3_O_4_.
3. Under cyclic load, the capsule forms microcracks on its outer surface through continuous contraction–expansion, and the internal chambers squeeze each other, resulting in the formation of pores on the chamber wall, and the healing agent inside the chamber discharges through the voids on the chamber wall and the cracks on the outer surface of the capsule. The capsule gradually discharges the rejuvenator under cyclic load. After 16,000, 32,000, 48,000, 64,000, 96,000, and 128,000 cycles of cyclic loading (0.7 MPa), the rejuvenator discharge rates of capsules are 23.9%, 36.4%, 44.8%, 49.5%, 56.7%, and 60.3%, respectively. The long-lasting and slow-discharge properties of the capsules are conducive to the realization of long-lasting repair of asphalt concrete.
4. Under the action of microwave irradiation, the magnetic nano-Fe_3_O_4_ particles embedded in the outer surface of the capsule rotate, vibrate, and exhibit other irregular movements, forming voids of varying sizes on the surface. Meanwhile, the nano-Fe_3_O_4_ particles on the wall of the internal chamber are subjected to irregular orientation movements in an alternating magnetic field, and at the same time, heat is generated, resulting in the emergence of micropores in the wall of the chamber, which prompts the restorative agent within the chamber to flow through the micropores and diffuse to the asphalt through the void on the outer surface. After 30, 60 s, and 90 s of microwave exposure, the rejuvenator discharge rates of capsule restorative are 31.7%, 46.2%, and 57.5%, respectively.

Previous studies have focused on the effects of calcium alginate capsules on the pavement performance, healing properties, and rheological properties of asphalt concrete and have not yet focused on the response mechanisms of the capsules under different external excitation conditions. This paper establishes an ideal model of the capsule’s internal chambers (pomegranate-like structure) and reveals the long-lasting release mechanism under cyclic loading and the active release mechanism under microwave irradiation of dual-responsive capsules. This paper offers a theoretical foundation for the all-season service of the dual-responsive capsules, which is anticipated to enable the long-term intelligent management of asphalt pavement. Future work will focus on targeted performance optimization of multi-responsive capsules based on pavement service conditions.

## Figures and Tables

**Table 1:** The essential features of raw materials.

Raw Material	Purity	Manufacture
Sodium alginate	CP	Sinopharm Chemical Reagent (Beijing, China)
Anhydrous CaCl_2_	CP	Sinopharm Chemical Reagent (Beijing, China)
Nano-Fe_3_O_4_ (50 nm)	99.9%	Chaowei nanomaterials Co., Ltd. (Shanghai, China).
Sunflower oil	Food grade	Arowana Group Co., Ltd. (Shanghai, China)
Tween 80	AR	Sinopharm Chemical Reagent (Beijing, China)

**Table 2:** The essential characteristics of sunflower oil.

Item	Value
Density (15 °C)	0.935 g/cm^3^
Viscosity (60 °C)	0.285 Pa·s
Flash point	230 °C

**Table 3:** The fundamental details of the instruments used in this study [39].

Experiment	Instrument	Manufacturers	Work Parameter
SEM	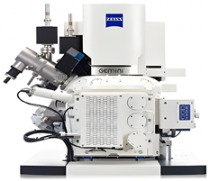	Gemini 300 Zeiss Oberkochen, Germany	Coat substance: Platinum powder Coat time: 30 s
UCT	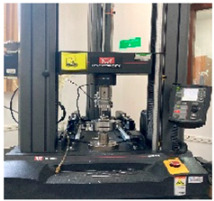	Instron 5967 Instron Norwood, MA, USA	Load speed: 0.05 mm/min
TGA	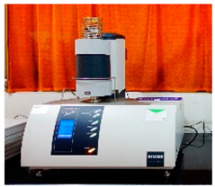	STA449F3 Netzsch Selb, Germany	Temperature range: 40~990 °C Temperature rise rate: 10 °C/min

**Table 4:** The gradation used in this work.

Sieve size/mm	16	13.2	9.5	4.75	2.36	1.18	0.6	0.3	0.15	0.075
Passing rate/%	100	96.5	78.2	51.9	33.3	21.0	14.6	11.2	9.0	6.8
Specific limit/%	100	90–100	68–85	38–68	24–50	15–38	10–28	7–20	5–15	4–8

## Data Availability

The original contributions presented in this study are included in the article. Further inquiries can be directed to the corresponding author.
